# The Application of an Iterative Structure to the Delay-and-Sum and the Delay-Multiply-and-Sum Beamformers in Breast Microwave Imaging

**DOI:** 10.3390/diagnostics10060411

**Published:** 2020-06-17

**Authors:** Tyson Reimer, Mario Solis-Nepote, Stephen Pistorius

**Affiliations:** 1 Department of Physics and Astronomy, University of Manitoba, Winnipeg, MB R3T 2N2, Canada; stephen.pistorius@umanitoba.ca; 2 Research Institute in Oncology and Hematology, University of Manitoba, Winnipeg, MB R3E 0V9, Canada; mario.solis.n@gmail.com

**Keywords:** microwave imaging, image reconstruction, breast imaging, microwave radar

## Abstract

Breast microwave imaging (BMI) is a potential breast cancer screening method. This manuscript presents a novel iterative delay-and-sum (DAS) based reconstruction algorithm for BMI. This iterative-DAS (itDAS) algorithm uses a forward radar model to iteratively update an image estimate. A variation of the itDAS reconstruction algorithm that uses the delay-multiply-and-sum (DMAS) beamformer was also implemented (the itDMAS algorithm). Both algorithms were used to reconstruct images from experimental scans of an array of 3D-printed MRI-based breast phantoms performed with a clinical BMI system. The signal-to-clutter ratio (SCR) and signal-to-mean ratio (SMR) were used to compare the performance of the itDAS and itDMAS methods to the DAS and DMAS beamformers. While no significant difference between the itDAS and itDMAS methods was observed in most images, the itDAS algorithm produced reconstructions that had significantly higher SMR than the non-iterative methods, increasing contrast by as much as 19 dB over DAS and 13 dB over DMAS. The itDAS algorithm also increased the SCR of reconstructions by up to 5 dB over DAS and 4 dB over DMAS, indicating that both high-intensity and background clutter are reduced in images reconstructed by the itDAS algorithm.

## 1. Introduction

Breast microwave imaging (BMI) has emerged as a potential breast cancer detection technique [1]. This modality uses non-ionizing microwave radiation to interrogate the breast tissues, and measurements of the resultant field produced after scattering from the tissues can be used to reconstruct an image of the breast. While X-ray mammography is the current breast imaging standard, its sizable false-positive rate (the cumulative risk of a false-positive after ten mammograms is estimated to be between 20–60% [2]), use of ionizing X-ray radiation and need for a highly trained operator make it less than ideal as a screening technique, particularly in remote rural communities. Because BMI systems use non-ionizing microwave radiation and are relatively low-cost, the modality has been proposed as a potential breast cancer screening method [1,3].

BMI systems use an antenna to illuminate the breast with a microwave signal. The tissues within the breast scatter this signal, and the resultant field can be measured using an antenna or array of antennas. The dielectric properties of the tissues govern the interaction of the microwave signal within the breast, and the contrast in the properties of malignant and healthy tissues [4,5] allows for the detection of malignant lesions by BMI systems.

Radar-based image reconstruction techniques in BMI aim to reconstruct a reflectivity map of the breast in which regions of strong reflections are prominently displayed. The primary reconstruction method in radar-based BMI is the delay-and-sum (DAS) beamformer [6]. This technique synthetically focuses the time domain signals collected by a radar system to reconstruct an image. Several derivatives of the DAS beamformer have been developed, including delay-multiply-and-sum (DMAS) [7], improved delay-and-sum (IDAS) [8], coherence-factor based delay-and-sum (CF-DAS) [9], and channel-ranked delay-and-sum (CR-DAS) [10]. The DAS-derivative algorithms have primarily been designed to reward coherence between measured radar signals [7,8,9] or account for path-dependent effects [10].

The DAS beamformer and its derivatives have been widely used and the image reconstruction method used in many patient imaging studies in radar-based BMI systems has been the DAS beamformer or its derivatives [11,12,13,14,15,16,17,18,19,20,21,22,23,24]. While other image reconstruction techniques have been proposed (microwave imaging via space time in [25,26] and data-adaptive methods in [27,28]), the DAS beamformer and its derivatives have been used as standards of comparison [29,30]. Recent patient and phantom investigations have found that only one DAS-derivative, the DMAS beamformer [7], reduced both background and high-intensity clutter while accurately localizing the tumor response in reconstructions [29,30]. 

This manuscript presents a novel iterative DAS-based reconstruction method. The functional form of the maximum-likelihood expectation-maximization (MLEM) algorithm used in positron emission tomography [31,32] is applied to the DAS beamformer. The iterative structure of the algorithm [31] was first presented for use in breast microwave radar imaging in a preliminary investigation [32]. The initial work in [32] did not apply the iterative algorithm to the DAS beamformer, and this investigation bridges the gap between the MLEM-based method described in [32] and the DAS method [6], resulting in the itDAS algorithm.

A second novel iterative algorithm is presented herein, in which the iterative structure is applied to the DMAS beamformer (resulting in the itDMAS algorithm). The iterative structure of the algorithm rewards signal coherence using multiple iterations and, through improvements to the forward signal model, allows for the modeling of path-dependent effects.

This work presents results on an array of anthropomorphic MRI-based breast phantoms (derived from the numerical models from [33]) that are representative of the four BI-RADS breast density classification categories [34]. The performance of the reconstruction methods as a function of breast density is discussed, and the results obtained with the iterative methods are compared to those obtained using the DAS and DMAS beamformers. To facilitate reproducibility and transparency, the code used to perform the analysis and produce the figures presented herein is open-source and available at Supplementary Materials https://github.com/TysonReimer/itDAS.

## 2. Methods

### 2.1. The itDAS Reconstruction Algorithm

**Figure 1:**
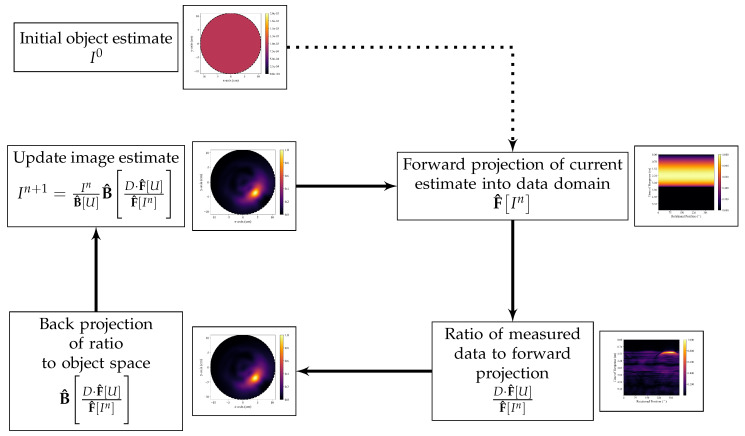
Flow chart of the functional form of the itDAS algorithm. The initial object estimate is forward-projected into the data domain, and the ratio between the measured data and this forward projection is determined. This ratio is then back-projected to the object space and the new image estimate is obtained by multiplying the previous image estimate by this back-projected ratio.

The iterative DAS-based algorithm presented herein is referred to as the itDAS algorithm. The itDAS beamformer adopts the iterative form of the MLEM reconstruction algorithm used in positron emission tomography (PET) [31]. The MLEM algorithm in PET is derived from the Poisson statistics of both the emission and detection events characteristic of PET [31]. This algorithm has a functional form that references only the forward and back projections of the imaging system. This functional form is displayed in Equation (1) with an additional normalization factor $\hat{F}\left[U\right]$ multiplying the dataset *D*,

(1) $$I^{n+1}=\frac{I^{n}}{\hat{B}\left[U\right]}\hat{B}\left[\frac{D\cdot \hat{F}\left[U\right]}{\hat{F}\left[I^{n}\right]}\right]$$

where $\hat{B}$ denotes the back-projection operator, $\hat{F}$ denotes the forward projection operator, *D* denotes the set of experimentally measured data, *U* is a unity matrix, and $I^{n}$ is the image estimate at the $n^{th}$ iteration. The back-projection operator transforms data from the data domain (in breast microwave radar imaging, this is the time domain) to the spatial domain of the image-space, and the forward projection operator simulates the dataset that would be obtained if the system scanned an object that would reproduce the image estimate. A flowchart of the functional form of the itDAS algorithm is displayed in Figure 1.

The iterative structure of Equation (1) was adopted for use in breast microwave radar imaging using the DAS beamformer as the back-projection operator. The initial image estimate was set to be a homogeneous map. For both the itDAS and itDMAS algorithms, the sixth iteration was the optimal balance of image contrast and noise and was selected as the stopping iteration.

A radar signal model, described in Equation (2), was used to compute the forward projection

(2) $$s_{m}\left(t\right)=\sum_{i=1}^{N}\sigma_{i}\delta \left(t-t_{m}\left(r_{i}\right)\right)$$

where $s_{m}$ is the radar signature measured at the $m^{th}$ antenna position, $\sigma_{i}$ is the reflectivity of the $i^{th}$ pixel, $r_{i}$ is the position of the $i^{th}$ pixel, and the sum is over all pixels in the object space. The time-of-flight $t_{m}$ is defined to be

(3) $$t_{m}\left(r\right)=2\frac{|r-r_{m}|}{v}$$

where *v* is the estimated average propagation speed of the signal in the object space, and **r**${}_{m}$ is the $m^{th}$ antenna position.

The DAS beamformer was used as the back-projection operator $\hat{B}$ in Equation (1). This method synthetically focuses the recorded radar signatures from each antenna position during a scan,

(4) $$I\left(r\right)=\sum_{m=1}^{M}s_{m}\left(t_{m}\left(r\right)\right)$$

where *I*(**r**) is the intensity value at point **r** in the reconstructed image, and the sum is over all *M* antenna positions used in the scan. After determining the intensity for each **r**, the reconstructed image is then presented as the squared intensity map.

### 2.2. The itDMAS Reconstruction Algorithm

While the itDAS algorithm uses the DAS beamformer as the back-projection operator, alternative techniques could be used. This work presents one alternative method, where the DMAS algorithm is used as the back-projection operator (resulting in the itDMAS algorithm). Two studies presented a comparison between radar-based reconstruction algorithms, evaluating six methods (the DAS, DMAS, IDAS, CF-DAS, CR-DAS, and robust capon beamformer [27] algorithms) using phantom data [30] and patient data [29]. The results in both studies indicate that among these algorithms, only the DMAS approach reduced both background and high-intensity clutter and accurately localized the tumor response [29,30].

The DMAS algorithm first performs antenna-pair multiplication before summation, rewarding coherence between measured signals. The reconstructed image is displayed as the square of the intensity map I(r),

(5) $$I\left(r\right)=\sum_{m=1}^{M-1}\sum_{n=m+1}^{M}s_{m}\left(t_{m}\left(r\right)\right)\cdot s_{n}\left(t_{n}\left(r\right)\right).$$

The itDMAS algorithm shares the functional form described in Equation (1) with the itDAS algorithm but uses the DMAS beamformer as the back-projection operator to further reward coherence between measured antenna-pair signals. In a monostatic radar system, this rewards coherence between signals measured at different antenna positions.

### 2.3. Validation with a Clinical BMI System and Breast Phantoms

A rotating radar-based BMI system [35] was used to perform experimental scans of anthropomorphic breast phantoms to evaluate the itDAS and itDMAS algorithms. The system uses a vector network analyzer (VNA) (Planar 804/1, Copper Mountain Technologies, Indianapolis, IN, USA) to generate a stepped-frequency continuous-waveform microwave signal over 1–8 GHz at 1001 frequency points. This signal is supplied to a double-ridged horn antenna (LB-20200-SF, A-INFO, Chengdu, China) that was used to illuminate the scan chamber with the microwave signal. The system operates in air, without the use of a coupling medium.

When performing a scan, the VNA recorded the S_11_ scattering parameters at 72 equally spaced positions along a circular trajectory centered at the axis of rotation of the imaging chamber. The S_11_ measurements were collected in the frequency domain, and the inverse chirp *z*-transform was used to convert the data to the time domain using 700 points between 0 ns and 6 ns. These time-domain signals were then used directly with the reconstruction approaches to produce images of the scanned phantoms. The measured frequency-domain S_11_ parameters for each phantom used in this investigation can be accessed at Supplementary Materials https://bit.ly/itDAS-data.

**Figure 2:**
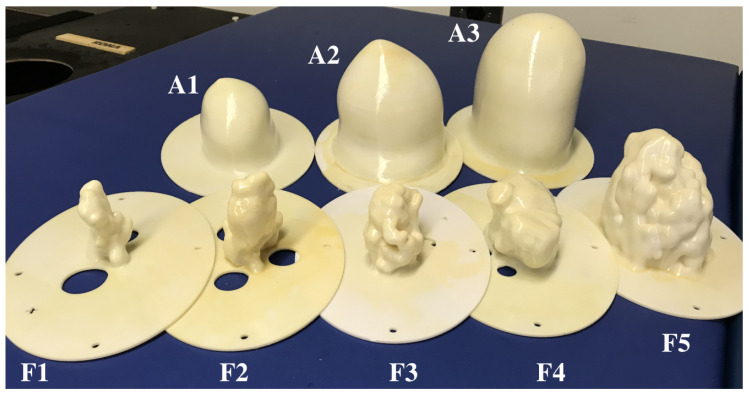
The array of 3D-printed MRI-derived breast phantoms. Adipose shells labeled A1 through A3, fibroglandular shells labeled F1 through F5.

An array of 3D-printed MRI-based breast phantoms were used in these experimental scans. The phantoms were derived from the publicly available phantom repository hosted by the University of Wisconsin-Madison [33] and were fabricated using the methods described in [36]. A photograph of the phantom array is displayed in Figure 2. The array consists of three adipose shells and five fibroglandular shells.

3D-printed phantoms allow for reproducible and controlled experimentation and have been used throughout the literature [33,36,37,38,39,40,41,42]. While the low-permittivity plastic layers are undesirable, they allow for the printing of morphologically accurate phantoms. To facilitate reproducibility, the 3D-printable .stl files used to produce the phantoms in Figure 2 are available at Supplementary Materials https://bit.ly/itDAS-phantoms.

When in use, a fibroglandular shell is inserted into an adipose shell. Each is then filled with a liquid designed to mimic the dielectric properties of the corresponding tissue. Glycerin was used to mimic adipose tissue, and a 30% Triton X-100 solution was used to mimic fibroglandular tissue [36]. Spherical glass bulbs of 15 mm and 10 mm radii filled with a saline solution were used as tumor analogs during the scans. The dielectric properties of the tissue analogs are described in [36].

Nine different adipose-fibroglandular shell combinations were experimentally scanned in this study. These phantoms are representative of all four of the BI-RADS breast density classification categories [34]. Table 1 displays the fibroglandular content by percent-volume of each of the phantom combinations used in this investigation. Eighteen scans were performed; each phantom was scanned once containing a 10 mm radius lesion and once containing a 15 mm radius lesion. The tumor analogs were positioned at the same height as the antenna (within an estimated uncertainty of 10 mm).

### 2.4. Skin Response Suppression and Propagation Speed Estimation

While the phantoms used in this investigation do not have a skin surrogate, large reflections occur at the air-plastic interface and at the plastic-adipose interface. Without the use of a coupling medium, the reflections at the air-phantom interface are sufficiently large to obscure the responses from the inner tissue structures.

Ideal skin suppression was used in this study to remove these air-tissue reflections and allow a direct comparison of the performance of the proposed iterative reconstruction methods and the literature standard non-iterative counterparts. Ideal suppression was implemented by performing two scans of each phantom. The first scan was performed with all phantom tissue components and the second scan was performed after removing the inner tissue components, leaving only the adipose surrogate. The difference between these two scans was used as the calibrated dataset.

An average propagation speed was used to compute the time-delays (in Equation (3)) for each beamformer. The permittivity in the breast was assumed to be that of the adipose tissue-mimicking material at the central scan frequency, ϵ = 6.4. The propagation speed used in the reconstruction algorithms was the average permittivity in the scan region assuming a circular breast. Accurate permittivity estimation in BMI is an active area of work [22,43,44], but the relatively straight forward approach of using an average estimated permittivity also allows for the direct comparison of the four reconstruction methods, without the influence of a sophisticated propagation speed correction, and has been used in other works comparing reconstruction methods [7,8,26,29].

### 2.5. Image Quality Metrics

Two image quality metrics were employed to quantitatively analyze the reconstructions produced by each of the four algorithms. These metrics were selected to measure the contrast present in an image. The signal-to-mean ratio (SMR) was defined as

(6) $$\mathrm{SMR}=20\log_{10}\left(\frac{S_{max}}{C_{mean}}\right)$$

where $S_{max}$ was the maximum response in the region known to belong to the tumor, and $C_{mean}$ was the mean response in the clutter region (defined to be the region of the image known to belong to the phantom, but not to the tumor). The region belonging to the tumor was assumed to be a circle of radius $r=r_{t}+5$ mm centered on the known tumor position, where $r_{t}$ was the radius of the spherical glass bulb used as the tumor surrogate. This was done to account for estimated positioning errors of the tumor during experimental scans. The signal-to-clutter ratio (SCR) was defined as

(7) $$\mathrm{SCR}=20\log_{10}\left(\frac{S_{max}}{C_{max}}\right)$$

where $C_{max}$ was the maximum response in the clutter region.

While no standard image quality metric is used throughout the literature, the definition of SMR in Equation (6) is the same as the definition of SCR in [37] and the same as in [45], and the definition of the SCR in Equation (7) is the same as in [45]. The SMR and SCR are measures of the contrast between the tumor response and the clutter in the image. The SCR provides a measure of the contrast between the tumor reflections and the strongest reflections in the image that belong to structures other than the tumor. A negative SCR indicates that the largest reflections in the image belong to a region outside of the known tumor location, while a large SCR indicates the tumor response is dominant in the reconstruction. A reconstruction with a negative SCR (indicating that the strongest response in the image was outside of the tumor location) was defined to be a reconstruction in which the tumor response was not identifiable.

While the SCR gives a measure of the contrast between the tumor response and high-intensity clutter, the SMR is a measure of the contrast between the tumor response and the average response within the breast region, indicating how easily the tumor response can be identified within the image with respect to the low-intensity clutter.

The localization error of the tumor response in reconstructions was also determined and was defined as the distance between the maximum-intensity response in the image and the center of the known tumor location.

To determine the uncertainty in the SMR and SCR, it was assumed that the uncertainty in $C_{mean}$ was zero and the estimated uncertainty in $S_{max}$ was defined to be the standard deviation of the intensities of the pixels with intensities within the 75th percentile of all pixels within the known tumor region. The estimated uncertainty in $C_{max}$ was defined to be the standard deviation of the intensities of the pixels with intensities within the 95th percentile of all clutter pixels.

## 3. Results and Discussion

### 3.1. Comparison of itDAS, itDMAS, DAS, and DMAS Methods

The DAS, DMAS, itDAS, and itDMAS beamformers were used to reconstruct images of each of the phantom scans. All beamformers produced 12 reconstructions that had identifiable tumour responses. For comparison, only these reconstructions are considered for analysis.

**Figure 3:**
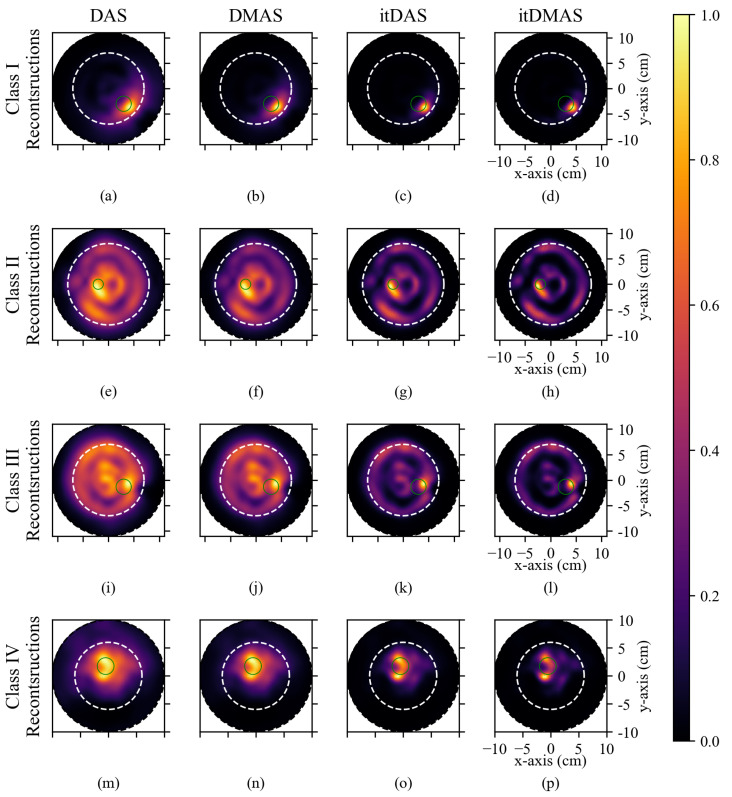
Reconstructions of breast phantoms by (left to right) the DAS, DMAS, itDAS, and itDMAS beamformers. (**a**–**d**) display the reconstructions of a 15 mm radius lesion in a Class I (A2F1) phantom, (**e**–**h**) display the reconstructions of a 10 mm radius lesion in a Class II (A3F4) phantom, (**i**–**l**) display the reconstructions of a 15 mm radius lesion in a Class III (A2F4) phantom, and (**m**–**p**) display the reconstruction of a 15 mm radius lesion in a Class IV (A1F4) phantom. Each image is normalized to its maximum intensity. The dotted black circle indicates the antenna trajectory during the scan, the dotted white line indicates the approximate breast phantom boundary, and the solid green circle indicates the known tumor position.

Figure 3 displays representative reconstructions produced by each algorithm for (a–d) a Class I phantom, (e–h) a Class II phantom, (i–l) a Class III phantom, and (m–p) a Class IV phantom. The reconstructed images were 2D coronal slices, at the same plane as the tumor location, and the approximate breast and tumor boundaries are indicated in the reconstructions. Table 2 displays the SMR and SCR for each reconstruction in Figure 3.

In all reconstructions, across all density classes, the itDAS and itDMAS algorithms produced images with higher SMR and SCR than either the DAS and DMAS methods, indicating that the iterative methods produce reconstructions with both reduced high-intensity and reduced low-intensity clutter, as shown in Figure 3. The iterative beamformers maintain the tumor response while significantly reducing the clutter intensity in the reconstructed images (as indicated in the SCR and SMR in Table 2).

The clinical system used to perform the scans of the breast phantoms operates in air without the use of a coupling medium. While ideal calibration of the air-tissue interface reflections was performed, the mismatch at the air-tissue interface results in clutter responses that are significant in the DAS and (to a lesser degree) the DMAS reconstructions.

All reconstructions of Class I phantoms prominently display the tumor response, as in Figure 3a–d. The iterative methods significantly reduced both the high-intensity clutter responses near the tumor location and the low-intensity background responses, compared to the non-iterative beamformers.

In the Class II and Class III reconstructions displayed in Figure 3e,f,i,j, the tumor response is not significantly greater than the high-intensity clutter responses (as indicated in the SCR in Table 2). The high-intensity clutter is present near the air-tissue boundary of the phantoms and near the center of the phantom. While the outer reflections are likely due to imperfect air-tissue reflection calibration, the central clutter response may be due to the fibroglandular tissues which are in the center of the phantoms.

The low contrast between this central response and the tumor response of the Class III reconstruction produced by both the DAS and DMAS methods; SCRs of (1 ± 1) dB and (2 ± 2) dB for the DAS and DMAS beamformers, respectively, makes the identification of the tumor response challenging. The itDAS and itDMAS reconstructions have higher SCRs; (4 ± 2) dB and (5 ± 3) dB, respectively.

While none of the reconstruction methods accurately reconstruct the lesion size, the tumor response is visible in the itDAS and itDMAS reconstructions and is localized within the expected region. No significant differences were observed in the localization error of the tumor response in the four examined reconstruction algorithms. The localization error of reconstructions produced by the DAS and DMAS methods was (11 ± 4) mm, (12 ± 5) mm for itDAS, and (11 ± 4) mm for itDMAS.

The average computation time required for image reconstruction with the itDAS algorithm was compared to that of the DAS and DMAS beamformers using a Ryzen 2700X central processing unit. When reconstructing 500 × 500 images (as presented in this article) from the 72 × 700 time-domain S_11_ array (700 time points per antenna position) used to represent each scan, the DAS algorithm required (13.1 ± 0.1) s to reconstruct an image, DMAS required (14.6 ± 0.2) s, and itDAS required (149 ± 2) s. While the ratio of computation time of itDAS compared to DAS was (11 ± 1):1, the total computation time was under 3 minutes and is not prohibitive.

itDAS requires the computation of forward and back projections at each iteration. The back projections are nearly equivalent to a complete DAS reconstruction with respect to computation time, requiring (11.9 ± 0.1) s. The forward projections required (9.1 ± 0.1) s. itDAS also requires the initialization of the forward and back projections of a unity dataset, $\hat{F}\left[U\right]$ and $\hat{B}\left[U\right]$ respectively. This initialization required (22.4 ± 0.1) s to complete. The majority of the computation time of itDAS is due to this initialization and the computation of the forward and back projections at each iteration.

The number of radar signals $N_{s}$ used to create the image is one factor that is expected to affect the algorithmic complexity of DAS-based reconstruction methods. As described in [46], DAS requires $N_{s}$ summations to reconstruct an image using $N_{s}$ measured radar signals, resulting in an algorithmic complexity of $O(N_{s})$ with respect to the number of measured radar signals used to produce the reconstruction. itDAS requires $N_{s}$ summations at each iteration to compute the back-projection and $N_{s}$ summations to compute the forward-projection, in addition to the 2$N_{s}$ summations required for the initialization of the forward and back projections of unity arrays. With respect to the number of measured radar signals, DAS has an algorithmic complexity of $O(N_{s})$ while itDAS has an algorithmic complexity of $O(2\left(N_{iter}+1\right)N_{s})$, where $N_{iter}$ is the number of iterations used to produce the reconstruction.

This model assumes that the summations used to compute the forward projections are the same as those used to produce the back projections, but the measured computation times indicate that forward-projection can be performed faster than back-projection. This model is therefore an overestimate of the algorithmic complexity of itDAS, but demonstrates that the algorithmic complexity is approximately linearly related to that of DAS with respect to the number of radar signals used for reconstruction. This model, in combination with the absolute and relative measured computation times stated above, demonstrate that while itDAS is more computationally complex and requires greater computation time to produce a reconstruction, these effects are not prohibitive.

**Figure 4:**
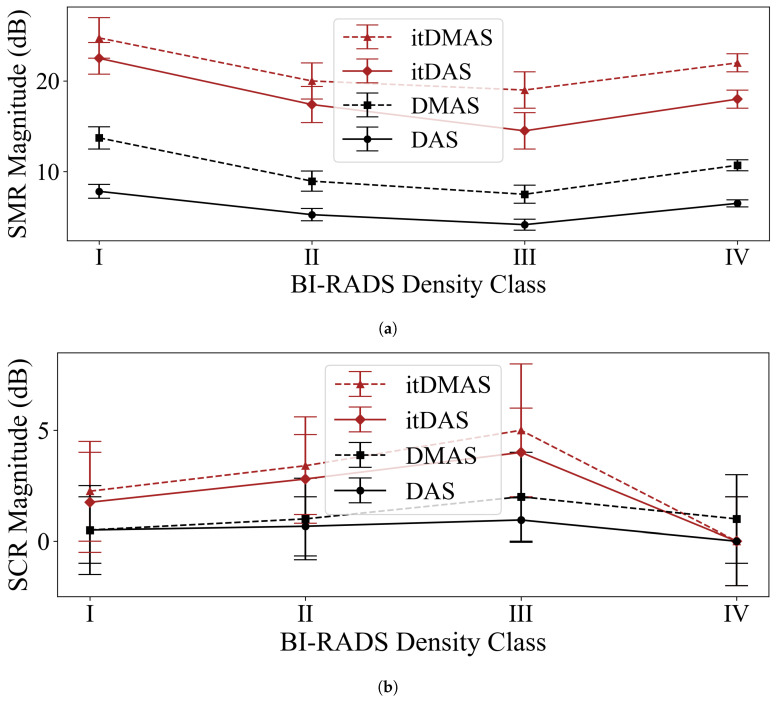
(**a**) Average SMR and (**b**) average SCR for the reconstruction algorithms using scans of phantoms representative of the four BI-RADS density classification categories.

Figure 4 displays the average SMR and SCR for reconstructions of each of the BI-RADS density classification categories with an identifiable tumor response (a reconstructed image was defined to have an identifiable tumor response if the SCR of the image was greater than zero) for each of the reconstruction algorithms. While a relatively small number of phantoms were used to represent each density classification category, the observed trend of decreasing SMR in higher-density breasts (excluding the Class IV phantom where only one of the four Class IV phantom reconstructions had an identifiable tumor response) agrees with the expected model of denser breasts presenting a more challenging reconstruction scenario for tumor detection.

This univariate analysis displays that in all density classes, the itDAS and itDMAS methods produced reconstructions with higher SMR and SCR than the DAS or DMAS beamformers. A multivariate analysis of contrast as a function of breast density, tumor size, tumor position, and breast size is outside the scope of this work, which aims to present the itDAS and itDMAS algorithms and make a comparison to the DAS and DMAS standards, but may be performed in future work using the phantom array.

As was found in phantom [30] and patient studies [29], images produced by the DMAS method consistently had higher SMR and SCR than reconstructions by the DAS algorithm. The DMAS beamformer produced reconstructions with an average increase in SMR of 75% relative to the DAS beamformer on Class I phantoms, 70% on Class II phantoms, and 81% on Class III phantoms, and 64% on Class IV phantoms.

While the DMAS reconstructions resulted in a 64–81% increase in SMR on average and a 0–111% increase in SCR on average, the iterative methods provided an even greater increase in contrast. The itDAS beamformer increased SMR by an average of 188% for Class I phantoms (relative to the DAS images), 231% for Class II, 249% for Class III, and 177% for Class IV. Improvements in SCR were as large as 321% for Class III phantoms. However, the itDMAS algorithm offered the greatest improvement over the DAS beamformer-increasing the SMR by as much as 358% in Class III phantoms and the SCR by 426% in Class III phantoms, on average.

The DMAS beamformer initially presented a significant improvement in clutter reduction in reconstructions for radar-based BMI [7] over the DAS beamformer [6]. The pair-wise signal multiplication rewards signal coherence while punishing incoherence [7]. Just as the DMAS method provided significant improvements over DAS, so too does the itDAS algorithm over DMAS. The multiplicative iterative structure further rewards signal coherence while punishing incoherence during reconstruction, resulting in images that prominently display tumor responses with significantly reduced low-intensity background clutter, and reduced high-intensity clutter. However, unlike the DMAS method, the itDAS and itDMAS algorithms have the potential to improve the forward signal model by accounting for path-dependent effects.

Both iterative algorithms resulted in significant increases in both SMR and SCR relative to the non-iterative beamformers, but the SCR and SMR of most reconstructions produced by the itDAS and itDMAS algorithms were not significantly different. This indicates that while the signal-pair multiplication in the DMAS beamformer rewards coherence between signals and results in higher contrast metrics in the DMAS images relative to the DAS images, the impact of this pairwise multiplication on the final reconstructed image is insignificant when combined with the effect of the iterative structure of Equation (1). The iterative structure is the dominant cause of the higher contrast present in the iterative reconstructions-for both the itDAS and itDMAS algorithms.

### 3.2. Future Work and Improving the Signal Model

Future work can investigate the impact of using other DAS-based methods as the back-projection operator in the iterative structure, including the IDAS [8], CF-DAS [9], and CR-DAS [10] methods. This investigation of the itDAS algorithm explored the use of the DMAS beamformer as the back-projection operator in the itDMAS algorithm due to its performance in [29,30], but alternative methods can also be explored.

Just as several DAS-derivative algorithms aimed to reward signal coherence [7,8,9], the iterative structure of the algorithm inherently rewards signal coherence (through multiplicative updates at each iteration) while punishing incoherence. While other DAS-derivatives have aimed at accounting for path-dependent effects [10] (and other radar-based reconstruction methods more broadly [26,27,32,37]), the itDAS algorithm is capable of both rewarding signal coherence and correcting for path-dependent effects through improvements to the forward model.

**Figure 5:**
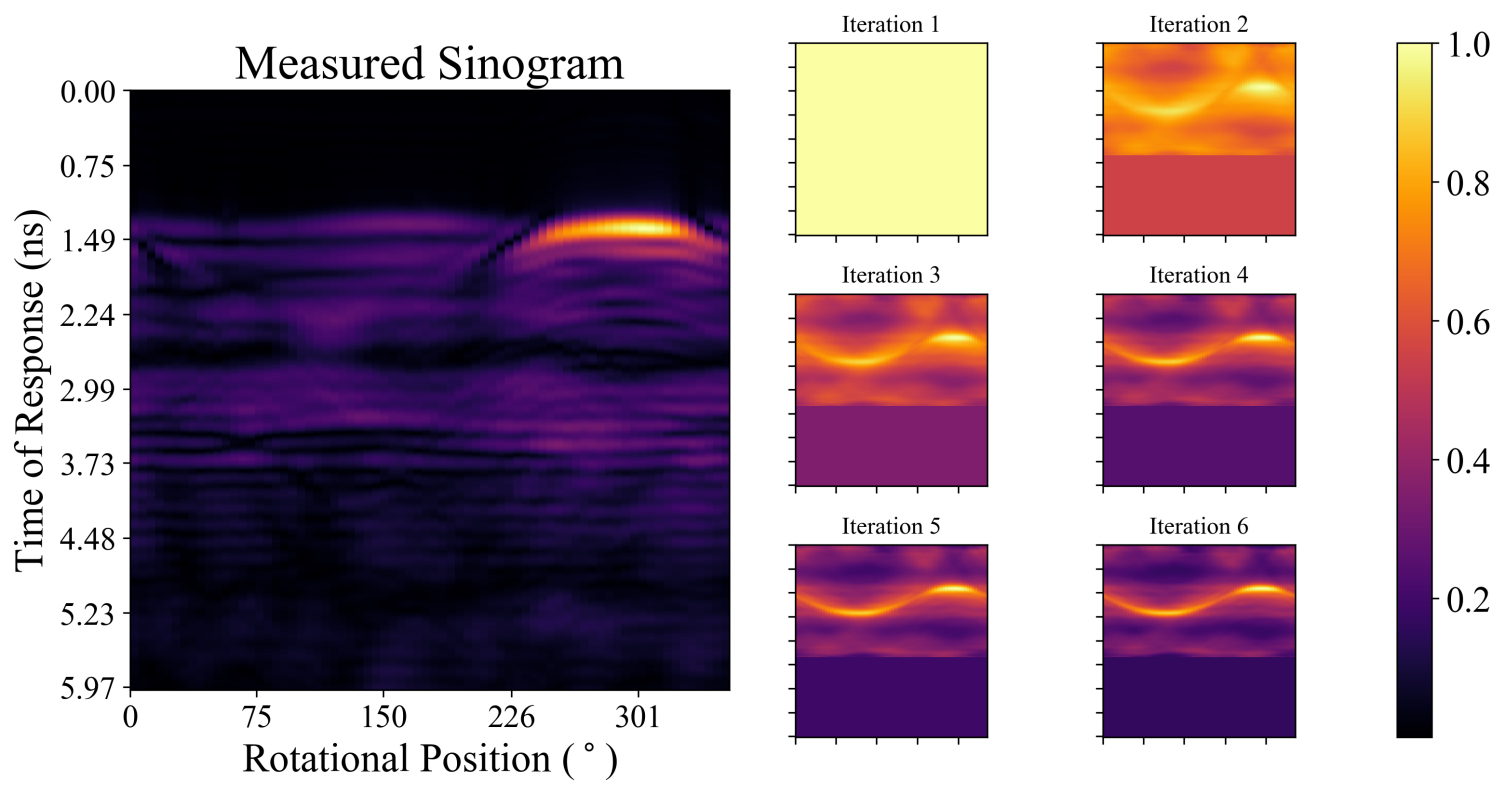
The measured sinogram (after ideal skin suppression) and the forward projection of the image estimate at each of the first six iterations during the itDAS reconstruction (displayed in Figure 3c) of the Class I phantom. Each sinogram and forward projection has been normalized to have a maximum value of unity.

The measured sinogram (after ideal skin suppression) for one of the phantom scans and the forward projection of the image estimate at each of the first six iterations during the itDAS reconstruction is displayed in Figure 5. While the tumour response is more apparent in later iterations, the differences between the forward projections and the measured sinogram highlight the limitations of the current radar signal model. Signal attenuation and inhomogeneous signal propagation affect the measured data but are not incorporated into the forward projection model presented herein.

Despite the limitations in the current forward projection model, the contrast improvements provided by the iterative structure of the itDAS method are significant, and the improvements in the reconstructions in Figure 3 are evident. The implementation of correction factors to improve the signal model used in the forward and back-projection operators will be explored in future work. Corrections for the antenna characteristics [32,37], the non-uniform propagation speed [37], and other path-dependent effects can be investigated. Previous work that had adapted the functional form of the MLEM algorithm from PET for use in BMI [32,37] found that the implementation of correction factors improved image contrast, and the impact of these corrections when this functional form is applied to the DAS beamformer will be investigated in future work.

The iterative structure of the itDAS algorithm requires the use of a stopping rule. The sixth iteration was selected as the final iteration for all reconstructions examined in this work, and future work should investigate more rigorous stopping rules based on phantom scans.

## 4. Conclusions

This work presents a novel iterative image reconstruction algorithm for use in breast microwave radar imaging. The presented itDAS method applies the functional form of the MLEM algorithm from PET [31] to the DAS beamformer [6], using the DAS beamformer as the back-projection operator and a radar signal model as the forward operator. A derivative of the itDAS algorithm using the DMAS method as the back-projection operator was also presented.

The DAS, DMAS, itDAS, and itDMAS algorithms were used to reconstruct images of eighteen tumor-containing experimental scans of 3D-printed MRI-based breast phantoms. In all cases, the itDAS and itDMAS algorithms produced images with higher SMR and SCR than either the DAS or DMAS methods.

The iterative structure of the itDAS algorithm inherently rewards signal coherence while punishing incoherence (through multiplicative image estimate updates) and has the potential to correct for path-dependent effects (through improvements to the forward model and modifications to the back-projection operator). Even without corrections for path-dependent effects, the itDAS algorithm produced reconstructions that had significantly higher SMR than the non-iterative methods, increasing contrast by as much as 19 dB over DAS and 13 dB over DMAS. The itDAS algorithm also increased the SCR of reconstructions by up to 5 dB over DAS and 4 dB over DMAS. No significant difference in the contrast of itDAS and itDMAS images was observed in the majority of reconstructions, indicating the rewarded coherence through the iterative structure is more significant than the effects of signal-pair multiplication present in the DMAS method. The promising results presented herein for an air-operated BMI system demonstrate the advantages of using the iterative beamforming methods.

## Figures and Tables

**Table 1:** Breast density and BI-RADS classifications of phantom combinations used in this manuscript.

	Fibroglandular Shell	Fibroglandular Volume	BI-RADS Classification
Small Adipose Shell (A1)	F1	4.4%	Class II
	F3	19.9%	Class III
	F4	37.2%	Class IV
Medium Adipose Shell (A2)	F1	1.8%	Class I
	F3	8.1%	Class II
	F4	15.0%	Class III
	F5	38.2%	Class IV
Large Adipose Shell (A3)	F2	2.7%	Class I
	F4	9.8%	Class II

**Table 2:** Image Quality Metrics of Reconstructions Displayed in Figure 3.

		DAS	DMAS	itDAS	itDMAS
Class I Images	SMR (dB)	11 ± 1	19 ± 2	29 ± 2	30 ± 3
	SCR (dB)	1 ± 2	1 ± 2	2 ± 3	2 ± 3
Class II Images	SMR (dB)	4.2 ± 0.5	7.7 ± 0.9	13 ± 2	16 ± 2
	SCR (dB)	0 ± 1	0 ± 2	1 ± 2	1 ± 2
Class III Images	SMR (dB)	3.2 ± 0.6	6 ± 1	12 ± 2	16 ± 2
	SCR (dB)	1 ± 1	2 ± 2	4 ± 2	5 ± 3
Class IV Images	SMR (dB)	6.5 ± 0.4	10.7 ± 0.6	18 ± 1	22 ± 1
	SCR (dB)	0 ± 2	1 ± 2	0 ± 2	0 ± 2

## Supplementary Materials

The Python code used to perform the analysis and prepare the figures contained in this manuscript is available at https://github.com/TysonReimer/itDAS. The data can be accessed at https://bit.ly/itDAS-data, and the .stl files used to 3D-print the phantoms can be accessed at https://bit.ly/itDAS-phantoms.
